# Prevalence of Nodding Syndrome — Uganda, 2012–2013

**Published:** 2014-07-18

**Authors:** Preetha J. Iyengar, Joseph Wamala, Jeffrey Ratto, Curtis Blanton, Mugagga Malimbo, Luswa Lukwago, Steven Becknell, Robert Downing, Sudhir Bunga, James Sejvar, Issa Makumbi

**Affiliations:** 1 EIS officer, CDC; 2 Uganda Ministry of Health; 3 Division of Global Health Protection, Center for Global Health, CDC; 4 Division of Global HIV/AIDS, Center for Global Health, CDC; 5 Division of High-Consequence Pathogens and Pathology, National Center for Emerging Zoonotic and Infectious Diseases, CDC

Nodding syndrome (NS) is a seizure disorder of unknown etiology, predominately affecting children aged 3–18 years in three sub-Saharan countries (Uganda, South Sudan, and Tanzania), with the primary feature of episodic head nodding. These episodes are thought to be one manifestation of a syndrome that includes neurologic deterioration, cognitive impairment, and additional seizure types. NS investigations have focused on clinical features, progression, and etiology (1–6); however, none have provided a population-based prevalence assessment using a standardized case definition. In March 2013, CDC and the Ugandan Ministry of Health (MOH) conducted a single-stage cluster survey to perform the first systematic assessment of prevalence of NS in Uganda using a new consensus case definition (7), which was modified during the course of the investigation. Based on the modified definition, the estimated number of probable NS cases in children aged 5–18 years in three northern Uganda districts was 1,687 (95% confidence interval [CI] = 1,463–1,912), for a prevalence of 6.8 (CI = 5.9–7.7) probable NS cases per 1,000 children aged 5–18 years in the three districts. These findings can guide the MOH to understand and provide the health-care resources necessary to address NS in northern Uganda, and provide a basis for future studies of NS in Uganda and in other areas affected by NS.

A sampling frame for the March 2013 assessment was provided by a house-to-house census conducted by village health teams (VHTs) in July 2012 in the northern Ugandan districts of Kitgum, Lamwo, and Pader, where most NS cases have been reported. During the census, VHTs asked the head of household whether anyone in that household has or ever had head nodding; 3,541 persons with reported head nodding were identified. Of these, 3,379 persons were eligible (162 had died) for participation in the 2013 assessment. A standardized questionnaire was used to further classify these cases based on the consensus case definition drafted at the first International Scientific Meeting on Nodding Syndrome in July 2012 (7). Respondents were asked about NS symptoms, epilepsy symptoms, development, cognitive functioning, medical history, and family history, and anthropometric measurements were taken. A suspected case was defined as reported head nodding (repetitive involuntary drops of the head towards the chest on two or more occasions) in a previously normal person. A probable case was defined as a suspected case with age of onset at 3–18 years and a frequency of nodding of 5–20 nods per minute, plus at least one of six minor criteria. A confirmed case was defined as a probable case with a documented nodding episode that was either observed and recorded by a trained health-care worker, videotaped, or documented with video electroencephalography or electromyography as atonic seizures (Table 1). The questionnaire was pilot-tested among other persons previously diagnosed with NS, children with epilepsy without head nodding, and healthy children.

To estimate the prevalence of NS, the target sample size was calculated assuming 50% of reported head nodding cases would be classified as probable NS with a CI of ±4.9%, a design effect of 1.5, and 10% nonresponse. With census survey data on number of reported head nodding cases per parish (a parish contains multiple villages), 30 parishes were selected by single-stage cluster sampling with probability proportional to size; 20–30 children with reported head nodding were selected per parish using simple random sampling without replacement. VHTs called selected persons and their caregivers to a central meeting point at a specified date and time. HCWs administered a standardized questionnaire in the local language supervised by CDC and MOH investigators. Results were weighted for unequal probabilities of selection and for nonresponse, and were adjusted for the projected age-sex distribution in the sampling frame. The denominator for the prevalence estimate was calculated using 2012 population projections based on 2002 Uganda Bureau of Statistics census data.

During pilot testing of the questionnaire, caregiver responses to questions about sexual development, a minor criterion in the consensus case definition, were judged unreliable for two main reasons. Caregivers were unable to consistently describe details of physical/sexual development of the children, and study teams were unable to independently verify “normal” versus “delayed” sexual development in the field. Therefore, this criterion was removed from the questionnaire before the assessment (Table 1).

A total of 767 persons with reported head nodding from the census were selected for the 2013 assessment; 23 were ineligible because they moved away, and 19 had died. Of the remaining 725 persons, 178 (24.6%) did not respond (four refused, and 174 were unavailable on the assigned interview date), for a response rate of 75% (547 of 725). Three were excluded from the analysis for incomplete survey responses. The median age of subjects was 13.9 years (range = 1.2–45.2 years); 53% were male. Available caregivers were interviewed for all participants; for 55% the interviewee was the mother, for 17% the father, and for 10% a grandmother.

Of the 544 included study subjects, 385 (71%) were reported to have a current or past history of head nodding. Of these, 325 (84%) were previously normal and therefore met the case definition for suspected NS. Because 121 (31%) gave responses of “don’t know” to questions regarding the major criterion of head nodding frequency, data quality was considered to be poor. Consequently, this criterion was dropped, and a modified case definition was used for the analysis. In the modified case definition, a suspected case of NS was the same as in the consensus case definition, but the probable case definition omitted the major criterion of head nodding frequency of 5–20 per minute and the minor criterion of delayed sexual development (Table 1). Of the 325 suspected cases, 300 met the major criterion of age of onset at ages 3–18 years and one minor criterion, and were considered probable cases by the modified case definition. The proportions that met the minor criteria varied, but no case met probable case definition that did not meet the minor criterion of clustering in space and time (Table 2). Of the 300 probable NS cases, 287 (96%) were in persons aged 5–18 years. For all age groups, the number of suspected NS cases was estimated to be 2,019 (CI = 1,758–2,281), with 1,834 (CI = 1,545–2,123) probable NS cases classified using the modified case definition in the three districts (Table 3). Among children aged 5–18 years, the number of suspected NS cases was estimated to be 1,782 (CI = 1,552–2,011), with 1,687 (CI = 1,463–1,912) probable NS cases according to the modified case definition, yielding a prevalence of 7.2 (CI = 6.3–8.1) suspected NS cases per 1,000 population and 6.8 (CI = 5.9–7.7) probable NS cases per 1,000 population in the three districts (Table 2) using the modified case definition.

## Discussion

Although an illness similar to NS has been reported in Tanzania since the 1960s, NS was only recently reported in South Sudan and Uganda. In 2009, the Ugandan MOH was notified of reports of possible NS cases in Kitgum District. These reports had apparently been increasing since 2003 (1), and few had been reported from other districts despite NS being widely publicized.

What is already known on this topic?

Nodding syndrome (NS) is a seizure disorder of unknown etiology that primarily affects children aged 3–18 years. It has been recognized in the sub-Saharan countries of Uganda, South Sudan, and Tanzania. Most investigations have focused on etiology and clinical progression; however, no population-based assessment of prevalence exists.

What is added by this report?

A consensus case definition was used for the first time to estimate prevalence of NS in northern Uganda. Using the modified consensus case definition developed during this study, the prevalence of probable NS among children aged 5–18 years in three districts in northern Uganda was estimated to be 6.8 cases per 1,000 children.

What are the implications for public health practice?

These results provide the most comprehensive assessment of the burden of NS in the region to date. The modified case definition can also be used in South Sudan and Tanzania to estimate the prevalence of NS in the affected regions of those countries. These data are needed to estimate the health-care resources necessary to support existing NS treatment centers in northern Uganda, and can be used as the basis for studies to establish mortality rates and treatment effectiveness to guide clinical care in other areas affected by NS.

Studies have demonstrated that NS is a seizure disorder with a sentinel and defining feature of paroxysmal episodes during which the head bobs forward repeatedly because of atonic seizures (1). A case series in Uganda demonstrated abnormal electroencephalographic (EEG) and brain magnetic resonance imaging findings in children with NS, confirming that it is a type of epilepsy, and primarily affects children aged 3–18 years (1). In South Sudan and Uganda, studies have indicated associations of NS with current or prior infection with the parasitic helminth *Onchocera volvulus* and vitamin B6 deficiency (4,8), but the etiology remains unclear. The mortality rate of NS is unknown, but deaths from injury similar to those associated with other forms of epilepsy have been reported (9). No proven effective treatment is available, but patients are empirically managed for their seizures with anti-epileptic medications.

The findings in this report are subject to at least four limitations. First, the census used for the sampling frame might have missed cases and resulted in an underestimation of NS prevalence. Second, the classification of NS, similar to epilepsy, relies on caregiver report, because self-report is not reliable and confirmatory techniques such as EEG are not always available (10). Such information is subject to misclassification and recall bias, especially when someone other than the primary caregiver is being interviewed. Third, NS questions used in this investigation were modeled on a previously validated epilepsy questionnaire (10); however, misclassification might still have resulted in over- or underestimation of NS cases and prevalence. Finally, nonresponse bias might have occurred because respondents were called to a meeting point, which might have excluded persons who were far away or unable to walk.

The prevalence of probable cases of NS was systematically assessed for the first time in the three northern Ugandan districts where most NS cases have been reported, and found to be 6.8 probable NS cases per 1,000 children. This investigation was the first to attempt to use the consensus case definition to determine prevalence (7). Modifications were necessary because certain criteria were difficult to assess based on caregiver recall or were not specifically defined. Also, the minor criterion of clustering alone did not clearly differentiate suspected from probable cases in this study population, but might be more useful in combination with other criteria or when used in other populations that have not already been screened. These data can inform future decisions on consensus case definition modifications. These results can also provide a basis for additional studies to establish mortality rates and treatment effectiveness, and for future studies in other areas affected by NS, such as South Sudan and Tanzania. This information is critical for guiding allocation of health-care resources to provide appropriate management of persons with NS in northern Uganda, and for designing a cohesive strategy to address this emerging public health problem in sub-Saharan Africa.

## Figures and Tables

**TABLE 1:** Consensus case definition and modified consensus case definition for nodding syndrome — Uganda, 2012–2013*

Type of case	Consensus case definition	Modified consensus case definition
**Suspected case**	Reported head nodding (repetitive involuntary drops of the head towards the chest on two or more occasions) in a previously normal person	Reported head nodding (repetitive involuntary drops of the head towards the chest on two or more occasions) in a previously normal person
**Probable case**	Suspected case of head nodding, with both major criteria: Age of onset of nodding ranging from 3 to 18 years Frequency of nodding 5–20 per minute	Suspected case of head nodding, with one major criterion: Age of onset of nodding ranging from 3 to 18 years
	Plus at least one of the following minor criteria: Other neurologic abnormalities (cognitive decline, school dropout because of cognitive or behavioral problems, other seizures or neurologic abnormalities) Clustering in space or time with similar cases Triggering by food or cold weather Stunting or wasting Delayed sexual or physical development Psychiatric symptoms	Plus at least one of the following minor criteria: Other neurologic abnormalities (cognitive decline, school dropout because of cognitive or behavioral problems, other seizures or neurologic abnormalities) Clustering in space or time with similar cases Triggering by food or cold weather Stunting or wasting Psychiatric symptoms
**Confirmed case**	Probable case, with documented nodding episode Observed and recorded by a trained health-care worker, or Videotaped nodding episode, or Video/EEG/EMG documenting head nodding as atonic seizures	Probable case, with documented nodding episode Observed and recorded by a trained health-care worker, or Videotaped nodding episode, or Video/EEG/EMG documenting head nodding as atonic seizures

**Abbreviations:** EEG = electroencephalographic; EMG = electromyographic.

* The consensus case definition was drafted at the first International Scientific Meeting on Nodding Syndrome, held July 30–August 1, 2012, in Kampala, Uganda. Meeting report available at http://www.who.int/neglected_diseases/diseases/Nodding_syndrom_Kampala_Report_2012.pdf. The modified consensus case definition was developed during the March 2013 single-stage cluster survey conducted by CDC and the Ugandan Ministry of Health to assess prevalence of nodding syndrome in Uganda.

**TABLE 2:** Number and percentage of suspected cases of nodding syndrome (N = 325) that met the minor criteria of the modified consensus case definition of a probable case, by selected criteria — Uganda, March 2013

Criteria	No.	(%)*
**Major criteria**		
Age of onset	300	(91)
**Minor criteria**		
Other neurologic abnormalities	321	(99)
Clustering in space and time	325	(100)
Triggering by food or cold weather	291	(88)
Stunting or wasting	83	(25)
Psychiatric symptoms	94	(28)

* Weighted percentage.

**TABLE 3:** Number of cases and prevalence of nodding syndrome, by age group — Uganda, March 2013

Age group	No. of cases assessed	Estimated no. of cases in all three districts covered	(95% CI)	Prevalence per 1,000 population	(95% CI)
**All ages (N = 650,800)**	544	3,379		—	
Reported head nodding	385	2,402	(2,152–2,651)	—	
Suspected cases	325	2,019	(1,758–2,281)	—	
Probable cases	300	1,834	(1,545–2,123)	—	
**Aged 5–18 yrs (248,243)**	489	2,913		—	
Reported head nodding	358	2,131	(1,925–2,338)	—	
Suspected cases	301	1,782	(1,552–2,011)	7.2	(6.3–8.1)
Probable cases	287	1,687	(1,463–1,912)	6.8	(5.9–7.7)

**Abbreviation:** CI = confidence interval.
